# Totally laparoscopic resection and natural orifice specimen extraction surgery (NOSES) in synchronous rectal and gastric cancer

**DOI:** 10.1093/gastro/goz064

**Published:** 2019-12-13

**Authors:** Yu-Liu-Ming Wang, Rui Huang, Hong-Yu Wu, Han-Qing Hu, Ying-Hu Jin, Qing-Chao Tang, Qiang Li, Wei-Yuan Zhang, Gui-Yu Wang, Xi-Shan Wang

**Affiliations:** 1 Department of Colorectal Surgery, The Second Affiliated Hospital of Harbin Medical University, Harbin, Heilongjiang, P. R. China; 2 Department of Colorectal Surgery, National Cancer Center/National Clinical Research Center for Cancer/Cancer Hospital, Chinese Academy of Medical Sciences and Peking Union Medical College, Beijing, P. R. China

## Introduction

Synchronous gastrointestinal cancer requires simultaneous resection. Recently, natural orifice specimen extraction surgery (NOSES) has become a new procedure of minimally invasive surgery [1]. Here, we report the first case of NOSES in the treatment of synchronous rectal and gastric cancer.

## Case report

The patient was a 68-year-old man with a past medical history of hypertension and coronary heart disease. The American Society of Anesthesiologists risk score was II and his body mass index was 28.1 kg/m^2^. The endoscopy examination found a 2-cm lesion in the gastric antrum and 3-cm lesion in the middle rectum. Pathological biopsy showed that both lesions were adenocarcinoma. No distant metastasis was found on computed tomography scan. No abnormal result was found in his laboratory examination. Preoperative staging of gastric cancer was cT2N0M0 (America Joint Committee on Cancer [AJCC] stage I) and rectal cancer was cT2N0M0 (AJCC stage I). A multidisciplinary treatment team assessed the patient’s general condition and agreed with preforming NOSES for simultaneous resection of the rectal and gastric cancer.

## Surgical procedure

### Preoperative preparation and pneumoperitoneum establishment

Gastrointestinal preparation and Nano-carbon labeling were performed 12 and 8 hours before surgery, respectively. After anesthesia, the patient was placed in the lithotomy position. A 10-mm trocar was inserted from the umbilicus for the laparoscope and two 5-mm trocars were inserted into the left-lower and right-lower quadrants. Two 12-mm working trocars were inserted into the left-upper and right-upper quadrants. The exploration of the abdominal cavity found no implantation or metastasis of the tumor.

### Laparoscopic gastrectomy

The gastric cancer was removed exclusively through laparoscopy; the common laparoscopic gastrectomy included subtotal gastrectomy, total omentectomy, D2 lymph-node dissection and Billroth II gastrojejunostomy [2, 3]. The specimen was put into a specimen bag and placed in the right side of the abdominal cavity.

### Laparoscopic dissection and establishment of the transanal route

For the rectal-cancer resection, we inserted another 12-mm trocar into the right-lower quadrant and another 5-mm trocar into the left-lower quadrant. Then, the patient was adjusted to the Trendelenburg position in order to expose the sigmoid colon, rectum, and inferior mesenteric artery [4]. The standard procedures for laparoscopic anterior resection consisted of a medial-lateral dissection, high ligation of the inferior mesenteric vessels with D3 lymph-node dissection, preservation of the autonomic nerves in the pelvic floor, and completion of the total mesorectal excision (TME) without pelvic side-wall dissection [4].

After dissection, one assistant flushed the rectum three to five times through the anus with a mixture of sterilized saline and iodophor before transecting the rectum. After adequate mobilization of the sigmoid colon and rectum, we transected the distal rectum at 3 cm below the tumor using the Endoscopic Linear Cutter-Straight. After that, we put a sterilized plastic protective sheath into the cavity through a 12-mm trocar and cut open the distal stump of the rectum with an ultrasound knife. Then, the assistant put an oval forceps head into the abdominal cavity through the anus and the rectal stump, and pulled out the sheath with oval forceps.

### Extraction of the gastric-cancer specimen

After establishing the transanal route, the assistant put the oval forceps head into the abdominal cavity again through the sterilized plastic protective sheath, caught up the specimen bag containing the gastric-cancer specimen, and extracted the specimen through the transanal route. The omentum was also extracted using the same method.

### Extraction of the proximal rectum and rectal-cancer resection

The proximal rectum (including the tumor) was extracted through the anus and the rectal stump. The rectum was pulled out of abdominal cavity through the transanal route and the following procedure was completed out of the cavity. We transected the rectum 5 cm above the tumor with purse-string forceps and knife, and then put an anvil into the lumen before purse-string suture. After sterilization, we put the stump of the proximal rectum (containing the anvil) back into the abdominal cavity.

### End-to-end colorectal anastomosis

The rectal opening was reclosed using another Endoscopic Linear Cutter-Straight. Then, end-to-end colorectal anastomosis was performed with a circular stapler using the double-stapling technique [5]. The seven holes of the trocar were closed after peritoneal lavage with the sterilized saline. The key steps of the procedures are shown in Figure 1.

### Post-operative outcomes

The operation time was 355 min and the blood loss was only 50 mL. The exhausted time was 22 hours and the ambulation time was 26 hours post procedure. The post-operative pathological result showed that the gastric cancer was an AJCC stage I (cT2N0M0) ulcerative moderately differentiated adenocarcinoma and the rectal cancer was an AJCC stage I (cT2N0M0) ulcerative well-differentiated tubular adenocarcinoma; the omentum was not invaded. The total number of lymph nodes examined for gastric cancer and rectal cancer was 18 and 13, respectively. All the incisal edges of the gastric cancer and the rectal cancer were not invaded. Complications including anastomotic leak and intra-abdominal abscess did not happen during hospitalization.

### Follow-up data

The patient did not receive adjuvant chemotherapy, since he had early-stage tumor. Five months post-operatively, the patient was in a healthy condition. In addition, the Holschneider score was used to assess the anal continence [6]. The preoperative score of this patient was 14 and the post-operative score was 12. These results showed that the anal continence was only slightly affected by the surgery.

## Discussion

Both the laparotomy and laparoscopic resection could not avoid wound-related complications. However, NOSES provides the opportunity to avoid abdominal incision and patients may recover more rapidly. Sumer *et al.* [2] reported the application of NOSES in the treatment of synchronous gastric and colon cancer, but we did not find any report of NOSES in the treatment of synchronous gastric and rectal cancer based on the research of PubMed and Web of Science.

It is important to assess aseptic and tumor-free principle for this new procedure. To assess the radical resection of cancer, the circumferential resection margin (CRM), distal margin, and the quality of the TME would be important markers. First, pathologists found that the involvement of the CRM was negative. The number of millimeters (mm) from the circumferential (radial) margin of the rectal cancer to the circumferential (radial) resection margin of the mesorectum was 8 mm. Second, the distal margin of the gastric cancer and rectal cancer was 55 and 30 mm, respectively. Besides, the quality of the TME specimen was considered as Grade 3, which represented a high-quality specimen. Finally, clamping the mouth of the specimen bag containing the gastric cancer and clamping the proximal rectum during extraction could avoid dissemination during NOSES. Accordingly, the NOSES procedure is in accordance with the tumor-free principle.

For assessment of the aseptic principle, complications including anastomotic leak and intra-abdominal abscess did not happen during hospitalization. Besides, the rectal lavage before transecting the rectum and peritoneal lavage before closing the trocar hole could be helpful. Finally, we put the sterilized plastic protective sheath into the cavity through a 12-mm trocar and pulled out the sheath through the rectal stump, which would be a unique method to avoid potential infection. Accordingly, our procedures could completely follow the aseptic and tumor-free principles.

In conclusion, we performed a simultaneous resection of gastric and rectal cancer using the NOSES procedure with acceptable operation time and little blood loss. The patient could recover more rapidly and no complications happened during hospitalization. Follow-up data showed that the patient was in a healthy condition. Thus, NOSES in the treatment of synchronous gastrointestinal cancer seems to be feasible and safe. However, more evidence and clinical research would still be needed to further explore the feasibility and safety of the NOSES procedure.

## Authors’ contributions

Y.L.M.W., G.Y.W., and X.S.W. designed the research; Y.L.M.W., R.H., H.Y.W., H.Q.H., Y.H.J., and Q.C.T. performed the research; Y.L.M.W., R.H., H.Y.W., Q.L., W.Y.Z., G.Y.W., and X.S.W. drafted the manuscript. All authors read and approved the final manuscript.

## Funding

This project was supported in part by grants from the National Natural Science Foundation of China [No. 81872034].
